# Benchmarking low- and high-throughput protein cleanup and digestion methods for human fecal metaproteomics

**DOI:** 10.1128/msystems.00661-24

**Published:** 2024-06-27

**Authors:** Alessandro Tanca, Maria Antonietta Deledda, Laura De Diego, Marcello Abbondio, Sergio Uzzau

**Affiliations:** 1 Department of Biomedical Sciences, University of Sassari, Sassari, Italy; 2 Unit of Microbiology and Virology, University Hospital of Sassari, Sassari, Italy; Pacific Northwest National Laboratory, Richland, Washington, USA

**Keywords:** FASP, gut microbiota, mass spectrometry, metaproteome, microplate, microtube, sample preparation

## Abstract

The application of fecal metaproteomics to large-scale studies of the gut microbiota requires high-throughput analysis and standardized experimental protocols. Although high-throughput protein cleanup and digestion methods are increasingly used in shotgun proteomics, no studies have yet critically compared such protocols using human fecal samples. In this study, human fecal protein extracts were processed using several different protocols based on three main approaches: filter-aided sample preparation (FASP), solid-phase-enhanced sample preparation (SP3), and suspension trapping (S-Trap). These protocols were applied in both low-throughput (i.e., microtube-based) and high-throughput (i.e., microplate-based) formats, and the final peptide mixtures were analyzed by liquid chromatography coupled to high-resolution tandem mass spectrometry. The FASP-based methods and the combination of SP3 with in-StageTips (iST) yielded the best results in terms of the number of peptides identified through a database search against gut microbiome and human sequences. The efficiency of protein digestion, the ability to preserve hydrophobic peptides and high molecular weight proteins, and the reproducibility of the methods were also evaluated for the different protocols. Other relevant variables, including interindividual variability of stool, duration of protocols, and total costs, were considered and discussed. In conclusion, the data presented here can significantly contribute to the optimization and standardization of sample preparation protocols in human fecal metaproteomics. Furthermore, the promising results obtained with the high-throughput methods are expected to encourage the development of automated workflows and their application to large-scale gut microbiome studies.

**IMPORTANCE:**

Fecal metaproteomics is an experimental approach that allows the investigation of gut microbial functions, which are involved in many different physiological and pathological processes. Standardization and automation of sample preparation protocols in fecal metaproteomics are essential for its application in large-scale studies. Here, we comparatively evaluated different methods, available also in a high-throughput format, enabling two key steps of the metaproteomics analytical workflow (namely, protein cleanup and digestion). The results of our study provide critical information that may be useful for the optimization of metaproteomics experimental pipelines and their implementation in laboratory automation systems.

## INTRODUCTION

The gut microbiota supports the human organism in numerous physiological processes, including food digestion and the immune response (1, 2). Alterations in the gut microbiota have been linked to the development of a range of chronic diseases, including metabolic dysfunctions, autoimmune disorders, inflammatory diseases, and cancer (3). Therefore, elucidating the composition and functions of the gut microbiota can provide crucial insights into human health and disease.

Human stool is a complex biological sample, containing a wide array of proteins of host, microbial, and dietary origin, along with many other biomolecules. It represents a good and non-invasive proxy of the colonic ecosystem, providing valuable insights into the host-microbiota relationship (4). In this regard, fecal metaproteomics offers the unique ability to simultaneously detect microbial and host protein functions and monitor their abundance variations in a taxon-specific fashion (5, 6).

While metaproteomics is being increasingly applied in biomedical studies, its successful implementation in large-scale investigations and clinical settings would require standardization of all the phases of the workflow, including sample collection and storage, sample preparation, liquid chromatography-tandem mass spectrometry (LC-MS/MS) analysis, and bioinformatics data analysis. Higher analytical throughputs, simpler and more reproducible experimental pipelines, and improvements in cost-effectiveness are among the most desirable advancements in sample preparation (7). To this end, the implementation of automated workflows using robotic workstations is expected to significantly improve the reproducibility and throughput of the metaproteomics analysis pipeline (8).

The effectiveness of a fecal metaproteomics sample preparation pipeline depends on three main steps: protein extraction, sample cleanup, and protein digestion. While multiplexing and automating the methods required to extract proteins from gut microbial communities in an efficient and unbiased manner still appear to be complex and technically demanding (9), fecal protein cleanup and digestion can be conveniently combined and scaled up to high-throughput, automated pipelines using multiwell microplates (10). In particular, fecal protein extracts contain numerous molecules (including detergents used for protein extraction) that can interfere with enzymatic digestion, chromatographic separation, and/or mass spectrometry detection, and therefore must be carefully removed (11). This goal can be achieved by several approaches, including protein precipitation and gel-based separation, which have been extensively applied to fecal samples (12, 13). However, these methods are labor-intensive and time-consuming, can suffer from operator-dependent reproducibility issues, and are inherently poorly suited for automation (14). Alternative protocols, that incorporate protein cleanup and digestion steps and are suitable for automation, are currently widely used in shotgun proteomics. These include filter-aided sample preparation (FASP) (15), solid-phase-enhanced sample preparation (SP3) (16), suspension trapping (S-Trap) (17), and in-StageTips (iST) (18). The incompatibility of the iST system with high SDS concentrations (such as those typically used for the extraction of proteins from complex microbial communities) makes it primarily applicable as an additional cleanup step in conjunction with other approaches, as exemplified by the SP3-iST combination. While FASP has been widely applied to human fecal protein extracts (19–23), only a few studies have described the use of S-Trap with this type of sample (10, 24). Furthermore, to the best of our knowledge, mouse stool is the only fecal sample type that has been processed using the SP3 method (25, 26). To date, no studies have been published that specifically compare human fecal protein cleanup and digestion protocols suitable for high-throughput applications.

In this study, we selected an array of methods for protein cleanup and digestion and compared them in both low-throughput (i.e., microtube-based) and high-throughput (i.e., microplate-based) formats, using human fecal protein extracts as input samples. More specifically, data-dependent LC-MS/MS and label-free quantification were employed to perform a comparative analysis of FASP-based (using filters with four different combinations of geometries and molecular weight cutoffs), SP3-based (followed or not by an additional iST purification), and S-Trap protocols. In addition, the efficiency of protein digestion, the ability to preserve hydrophobic peptides and high molecular weight proteins, and the reproducibility of the methods were evaluated.

## MATERIALS AND METHODS

### Sample collection and protein extraction

The fecal samples used in this study were obtained as part of a larger collaborative study (27) that was approved by the Institutional Review Board of the local Ethics Committee (ASL n. 1 Sassari, Prot. No. 2358/CE). All subjects provided written consent for the use of their fecal material for research purposes. The 12 subjects who provided the stool samples used in this study were enrolled after a diagnosis of *Helicobacter pylori* infection and collected their samples 30 days after the end of a successful antibiotic treatment, which resulted in complete eradication of the infection. The subjects were 10 females and two males, and their ages ranged from 40 to 72 years (with a mean of 52 years). The fecal samples were collected using a sterile disposable stool container, delivered to the laboratory within 2 hours, and stored at −80°C until further processing.

At the time of protein extraction, all fecal samples were thawed at 4°C. A fragment of approximately 150 mg was collected from each fecal sample and placed in a microtube. Proteins were extracted according to established procedures (28), with minor modifications. The extraction buffer (2% SDS, 100 mM DTT, 20 mM Tris-HCl pH 8.5; 100 µL per 50 mg of stool) and a steel bead (5 mm diameter; Qiagen, Hilden, Germany) were added to each sample. Each microtube, containing a fecal fragment, the extraction buffer, and a bead, was then processed as follows: incubated at 95°C for 20 min in a thermoblock (FALC, Treviglio, Italy), incubated at −80°C for 10 min, subjected to bead beating for 10 min (30 cycles/s in a TissueLyser LT mechanical homogenizer, Qiagen), incubated at −80°C for 10 min, incubated at 95°C for 10 min, subjected to bead beating for 10 min (30 cycles/s), and centrifuged at 14,000 × *g* for 10 min. The final supernatants were collected and stored at −20°C until further processing.

### Low-throughput protein cleanup and digestion methods

**Fig 1:**
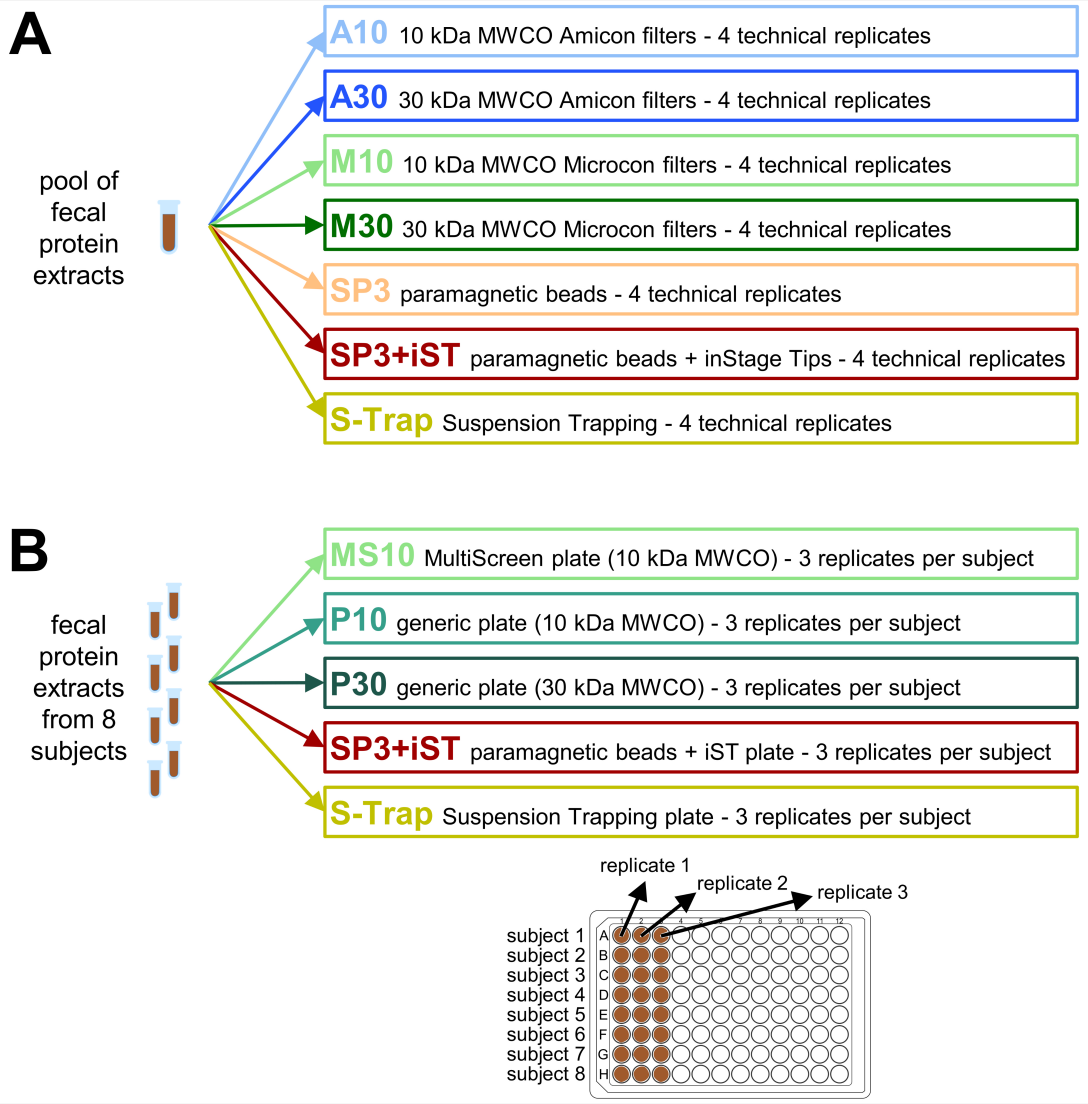
Experimental design of the study. (**A**) Comparison of low-throughput (microtubes) sample preparation methods. (**B**) Comparison of high-throughput (microplate) sample preparation methods. iST, inStage Tips; MWCO, molecular weight cutoff; SP3, single-pot, solid-phase-enhanced sample preparation.

For the comparison of low-throughput methods (i.e., performed in microtubes), a pool was prepared by mixing 200 µL of protein extracts from four different volunteers. The pooled proteins were then subjected to alkylation by adding 80 µL of 200 mM iodoacetamide to the solution and incubating for 20 min in the dark at room temperature. Subsequently, 28 identical 20 µL aliquots were collected from the pooled protein mixture, thus enabling the analysis of four technical replicates for each of the seven low-throughput sample preparation methods being compared, as shown in Fig. 1A.

The first four methods were all based on the filter-aided sample preparation (FASP) protocol (15, 29). The following four types of centrifugal filters were employed (all from Merck, Darmstadt, Germany): Amicon 10 kDa Ultra-0.5 centrifugal filter units with Ultracel-10 membrane (A10), Amicon 30 kDa Ultra-0.5 centrifugal filter units with Ultracel-30 membrane (A30), Microcon 10 kDa centrifugal filter units with Ultracel-10 membrane (M10), and Microcon 30 kDa centrifugal filter units with Ultracel-30 membrane (M30). The aliquots of the protein mixture were diluted with 200 µL of dilution buffer (8 M urea in 20 mM Tris-HCl, pH 8.5), loaded into the centrifugal filters, and centrifuged at 14,000 × *g* for 10 or 20 min (for 30 kDa and 10 kDa cutoff filters, respectively). Three sequential washing steps were then performed with 200 µL of dilution buffer, 100 µL of 50 mM ammonium bicarbonate, and another 100 µL of 50 mM ammonium bicarbonate, each followed by centrifugation at 14,000 × *g* for 10 or 20 min (for 30 kDa and 10 kDa cutoff filters, respectively). Next, 400 ng of trypsin (from porcine pancreas, lyophilized powder, Proteomics Grade, BioReagent, dimethylated, purchased from Sigma-Aldrich, now part of Merck), resuspended in 100 µL of 50 mM ammonium bicarbonate, was added to each sample. The samples were then mixed for 1 min at 500 rpm in a thermomixer (Fisherbrand Isotemp Cooling Shake Touch Shaker, Thermo Fisher Scientific, Waltham, USA) and incubated at 37°C for 18 h. After centrifugation at 14,000 × *g* for 10 or 20 min (for 30 kDa and 10 kDa cutoff filters, respectively), 100 µL of elution solution (20% acetonitrile, 0.2% formic acid) was added to the samples, which were then centrifuged again at 14,000 × *g* for 10 or 20 min (for 30 kDa and 10 kDa cutoff filters, respectively). The eluates were collected and transferred to clean tubes.

The fifth method was based on the single-pot, solid-phase-enhanced sample preparation (SP3) protocol (30). The SP3-iST (8rxn) Add-on kit (PreOmics, Martinsried, Germany), containing paramagnetic beads and related solutions, was purchased and used according to the manufacturer’s instructions. The beads were resuspended by vortexing, washed twice with the “resuspend” solution, using a magnetic separator (as in all the subsequent steps), and finally resuspended in 160 µL of “resuspend” solution. Subsequently, 20 µL of beads were added to each aliquot of protein mixture, followed by 120 µL of “bind” solution and incubation in a thermomixer at 1,000 rpm and room temperature for 15 min. Samples were washed three times with 150 µL of “wash” solution. Afterward, 1.6 µg of trypsin (of the same type, manufacturer, and lot used for the previous methods), resuspended in 100 µL of 50 mM ammonium bicarbonate, was added to each tube. The samples were incubated in a thermomixer at 37°C and 1,000 rpm for 3 hours. Subsequently, 100 µL of “stop” solution was added, and the samples were mixed. Centrifugation at 20,000 × *g* at 24°C for 1 min was then performed, after which the supernatants were collected and transferred to clean tubes.

The sixth method was a sequential combination of the SP3 and inStage Tip (iST) (18) protocols. To this end, two kits from PreOmics were employed: the above-mentioned SP3-iST (8rxn) Add-on kit and the iST 8× kit. The first part of the method was identical to the SP3 protocol described above (until the addition of the “stop” solution). Then, according to the manufacturer’s instructions, the samples were transferred to the iST cartridges and centrifuged at 3,800 × *g* for 2 min. Two sequential washing steps were then performed, with 200 µL of “wash 1” and “wash 2” solutions, respectively. Each washing step was followed by centrifugation at 3,800 × *g* for 2 min. Subsequently, 100 µL of “elute” solution was added, and the eluates obtained following centrifugation at 3,800 × *g* for 2 min were collected and transferred to clean tubes.

The seventh method was the suspension trapping (S-Trap) protocol (17), which was carried out using the S-Trap micro kit from ProtiFi (Farmingdale, USA), according to the manufacturer’s instructions. As a preliminary step, 1.2 µL of SDS 10% was added to each aliquot of the protein mixture to reach an SDS concentration of 2%. Phosphoric acid (final concentration of 2.75%) and 165 µL of “buffer 5” supplemented with methanol were then added sequentially to each sample. Following this, the samples were mixed, transferred to the S-Trap columns, and centrifuged at 10,000 × *g* for 30 s. Three sequential washing steps with “buffer 5” were then performed, each followed by centrifugation at 10,000 × *g* for 30 s. The S-Trap columns were transferred to clean tubes, and 4 µg of trypsin (of the same type, manufacturer, and lot used for the previous methods), resuspended in 20 µL of 50 mM ammonium bicarbonate, was dispensed into each column. Following incubation for 18 h at 37°C, three sequential steps were carried out by adding 40 µL of 50 mM ammonium bicarbonate, 40 µL of 0.2% formic acid, and 40 µL of 50% acetonitrile, respectively. Each step was followed by centrifugation at 10,000 × *g* for 1 min. The eluates were collected and transferred to clean tubes.

Finally, the peptide mixtures (*N* = 28) were dried using a Concentrator Plus (Eppendorf, Hamburg, Germany) and shipped to an external laboratory for LC-MS/MS analyses.

### High-throughput protein cleanup and digestion methods

The eight remaining protein extracts, each from a different subject, were used to compare the high-throughput (i.e., performed in 96-well microplates) methods. For each subject, 350 µL of protein mixture was alkylated by adding 35 µL of 200 mM iodoacetamide to the solution, followed by incubation for 20 min in the dark at room temperature. Fifteen identical 20 µL aliquots were then prepared from each protein mixture, thus enabling the analysis of three technical replicates for each of the five high-throughput sample preparation methods being compared, as shown in Fig. 1B. Given the geometry of the 96-well microplate (with 8 rows and 12 columns), the protein mixtures from different volunteers were loaded in different rows, whereas the three technical replicates from the same volunteer were loaded in adjacent columns on the same row.

The first three methods, all based on the FASP protocol, were performed using two different types of microplates, namely the MultiScreen 96-well Ultrafiltration Plate with Ultracel-10 membrane kD (Merck; MS10) and the AcroPrep Advance 96-well Filter Plate for Ultrafiltration (Pall, Port Washington, USA). The latter microplate was tested in two variants, with a molecular cutoff of 10 kDa (P10) or 30 kDa (P30). A standard receiver microplate was placed under each filtering microplate. Each aliquot of the protein mixtures was diluted with 380 or 280 µL (for MS10 and P10/P30, respectively) of dilution buffer (8 M urea in 20 mM Tris-HCl, pH 8.5) and loaded in a separate well of the microplates. The microplates were then centrifuged at 2,500 × *g* for 20 or 8 min (for MS10 and P10/P30, respectively). Subsequently, three sequential washing steps were performed with 200 µL of dilution buffer, another 200 µL of dilution buffer, and 200 µL of 50 mM ammonium bicarbonate, each followed by centrifugation at 2,500 × *g* for 20 or 8 min (for MS10 and P10/P30, respectively). Subsequently, 400 ng of trypsin (the same type and manufacturer described for low-throughput methods), resuspended in 100 µL of 50 mM ammonium bicarbonate, was added to each sample. The samples were then incubated at 37°C with agitation for 18 h. After centrifugation at 2,500 × *g* for 20 or 8 min (for MS10 and P10/P30, respectively), 100 µL of elution solution (20% acetonitrile, 0.2% formic acid) was added to the samples, which were centrifuged again at 2,500 × *g* for 20 or 8 min (for MS10 and P10/P30, respectively). The eluates were collected in a clean receiver microplate and then transferred to clean tubes.

The fourth method involved a sequential application of SP3 and iST protocols, whereby the commercial kits SP3-iST (96rxn) and iST 96× (PreOmics) were combined in accordance with the manufacturer’s instructions. Bead resuspension and washing were performed according to the procedure described for the low-throughput SP3 protocol. The aliquots of the protein mixtures were loaded in the wells of a standard low-binding 96-well microplate, with 20 µL of beads added to each sample. This was followed by the addition of 120 µL of “bind” solution and incubation in a shaker for 15 min. The samples were then washed three times with 150 µL of “wash” solution, using a magnetic separator suited for 96-well microplates. Subsequently, 1.6 µg of trypsin (of the same type, manufacturer, and lot used for the previous methods), resuspended in 100 µL of 50 mM ammonium bicarbonate, was added to each well. The samples were then incubated at 37°C with agitation for 3 hours. After the incubation, 100 µL of “stop” solution was added. The samples were then mixed and transferred to the iST cartridges (placed on top of the “waste” plate provided with the kit) and centrifuged at 2,250 x *g* for 2 min. Two sequential washing steps were performed, each with 200 µL of “wash 1” and “wash 2” solutions, respectively. Each washing step was followed by centrifugation at 2,250 x *g* for 2 min. After transferring the iST cartridges to the “collection” plate provided with the kit and adding 100 µL of “elute” solution, the eluates obtained following centrifugation at 2,250 x *g* for 2 min were collected and transferred to clean tubes.

The fifth high-throughput method was carried out using the S-Trap 96-well plate from ProtiFi, in accordance with the manufacturer’s instructions. As a preliminary step, 1.2 µL of SDS 10% was added to each protein mixture sample to reach an SDS concentration of 2%. After placing the S-Trap microplate atop a standard receiver microplate, phosphoric acid (final concentration of 1.2%) and 350 µL of binding buffer (90% methanol, 100 mM ammonium bicarbonate, pH 7.1) were added sequentially to the samples. The samples were mixed and transferred to the wells of the S-Trap microplate. The microplate was then centrifuged at 1,500 x *g* for 2 min. Thereafter, three sequential washing steps with 200 µL of binding buffer were performed, each followed by centrifugation at 1,500 x *g* for 2 min. A clean receiver plate was then placed beneath the S-Trap microplate, and 1.6 µg of trypsin (of the same type, manufacturer, and lot used for the previous methods), resuspended in 125 µL of 50 mM ammonium bicarbonate, was dispensed into each well. Subsequently, the samples were incubated at 47°C for 1 h. Three sequential steps were then carried out by adding 80 µL of 50 mM ammonium bicarbonate, 80 µL of 0.2% formic acid, and 80 µL of 20% acetonitrile, 0.5% formic acid solution, respectively, each followed by centrifugation at 1,500 x *g* for 2 min. The eluates were collected in the receiver microplate and then transferred to clean tubes.

Finally, the peptide mixtures (*N* = 120) were dried using a Concentrator Plus and shipped to an external laboratory for LC-MS/MS analyses.

### LC-MS/MS analyses

LC-MS/MS analyses were performed in service at the Laboratory of Proteomics-UMG of the University of Catanzaro (Italy). Offline StageTip purification and nanoLC analysis were performed as described elsewhere (31). Reconstituted peptide mixtures (approximately 5 µg) were purified using SCX StageTips, eluted in 10 µL of 500 mM ammonium acetate containing 20% acetonitrile, then evaporated to dryness and resuspended in 0.2% formic acid. A 200 ng aliquot of each peptide mixture was injected for a preliminary nanoLC-MS/MS analysis. The appropriate injection volume (between 1 and 8 µL) was assessed by analyzing a 1 µl aliquot of each peptide mixture using a short LC gradient and then estimating the peptide amount based on the total peptide signal (area under the curve). LC was performed on an EasyLC 1200 instrument (Thermo Fisher Scientific). The nanoLC column was a 0.075 × 160 mm (internal diameter and column length, respectively) pulled capillary, packed in-house with C18 silica particles (Dr. Maisch, Ammerbuch, Germany). The peptides were loaded into mobile phase A (2% acetonitrile, 0.1% formic acid) at a flow rate of 500 nL/min and eluted at 300 nL/min by the following gradient: from 0% B to 25% B (80% acetonitrile, 0.1% formic acid) in 60 min, from 25% B to 45% B in another 10 min, and then to 100% B in 8 min. The column was regenerated for 10 min at 100% B and equilibrated at 0% B for 20 min prior to the next injection. Two blank injections were performed between samples. A shorter gradient (45 min) was utilized for blank injections.

Peptides were electrosprayed in a positive ion mode into an Orbitrap Exploris 480 (Thermo Fisher Scientific), using 1800 V as spray voltage. Internal calibration was automatically performed at the beginning of each run (RunStart Easy IC-on). The full scan MS parameters were as follows: scan range, 375–1400 m/z; resolution, 60,000; RF lens, 40%; AGC target, 100%; maximum injection time, 50 ms. Data-dependent acquisition was performed using the following parameters: dependent scans, 15 (top-15); dynamic exclusion, 20 sec; charge states, 2–6; and intensity threshold, 5e^4^. Tandem mass spectrometry scans were acquired according to the following parameters: isolation window, 1.6 m/z; resolution, 30,000; normalized collision energy, 30%; AGC target, 100%; and maximum injection time, 120 ms.

### Bioinformatic analyses

Peptide identification was performed using Proteome Discoverer™ software (v.2.5; Thermo Fisher Scientific), with Sequest-HT as the search engine and Percolator for peptide validation, setting the false discovery rate (FDR) threshold to 1%. The search parameters were as follows: precursor mass range, 350–5000 Da; minimum peak count, 6; S/N threshold, 2; enzyme, trypsin (full); maximum missed cleavage sites, 2; peptide length range, 5–50 amino acids; precursor mass tolerance, 10 ppm; fragment mass tolerance, 0.02 Da; static modification, cysteine carbamidomethylation; and dynamic modification, methionine oxidation. Searches were conducted in parallel against two sequence databases: a collection of human gut metagenomes (available at https://ftp.cngb.org/pub/SciRAID/Microbiome/humanGut_9.9M/GeneCatalog/IGC.pep.gz) (32) and the *Homo sapiens* reference proteome retrieved from UniProtKB/Swiss-Prot (release 2021_04). Proteins were categorized as “microbial” or “human” when they belonged to the first or second database, respectively. Proteins were grouped by the Proteome Discoverer algorithm according to the strict parsimony principle, selecting a representative master protein for each protein group. Peptide-spectrum match (PSM) data were also exported to extrapolate posterior error probability (PEP) values calculated by Percolator.

Offline mass recalibration and label-free MS1 quantitation were carried out using the Spectrum Files RC and the Minora Feature Detector nodes, respectively (33). The optimal settings for retention time and mass tolerance windows were calculated by the Minora algorithm based on mass accuracy and retention time variance distribution. A consensus feature list was defined based on the outputs of the Feature Mapper and Precursor Ions Quantifier nodes. MS1 signals of all peptides exhibiting significant matches with at least one MS2 spectrum from at least one sample were mapped across runs and quantified by calculating the integrated area of the chromatographic peak. Master protein abundances were calculated as the sum of the abundances of all peptides matching that protein sequence.

Unipept Desktop (v.2.0.0) was used to carry out peptide taxonomic annotation (34), selecting the three available options (“equate I and L,” “filter duplicate peptides” and “advanced missed cleavage handling”). Protein sequences were subjected to functional annotation using the eggNOG-mapper web application (v.2.1.9, available at http://eggnog-mapper.embl.de/) (35), keeping default parameters and then choosing KEGG (Kyoto Encyclopedia of Genes and Genomes) orthology (KO) information as main functional classification (36). Meta4P (v.1.5.3) was employed to parse identification, quantification, and annotation data, as well as to calculate the related metrics (37). The grand average of hydropathy (GRAVY) score was calculated for each peptide sequence identified in the study using the online GRAVY calculator (https://www.gravy-calculator.de).

### Statistical analyses and graph generation

A statistical comparison between sample groups (i.e., different methods) was conducted using one-way ANOVA, Tukey’s correction for multiple testing, and an adjusted *P*-value < 0.05 as the significance threshold, as implemented in GraphPad Prism (v.9.0.0). Specifically, ordinary ANOVA was employed for low-throughput methods data (comprising independent technical replicates), whereas repeated measures ANOVA with Geisser-Greenhouse correction was employed for high-throughput method data to perform within-subject comparisons (starting from means of replicate values for each subject-method combination). Spearman’s correlation analysis was conducted using the R package *Hmisc*. Scatter plots and violin plots were created using GraphPad Prism (v.9.0.0). Principal component analysis (PCA) plots were generated using the ClustVis web application (38), using the default analysis parameters. Peptide abundance data were used as input for PCA, with peptides that had not been quantified in at least 4 and 60 samples for low- and high-throughput methods data, respectively, being excluded from the analysis. Principal component scores were loaded into PRIMER 7 (v.7.0.23) to calculate Euclidean distances between samples. Finally, PERMANOVA was applied to the distance values (with sequential sum of squares and 999 permutations as parameters) to test the significance of the separation between sample groups.

## RESULTS

### Comparison of low-throughput sample preparation methods

In the first part of the study, a comparative experiment was designed by selecting seven established low-throughput (i.e., to be performed in microtubes) protein cleanup and digestion protocols and a pool of human fecal protein extracts as the sample. As schematized in Fig. 1A, four methods were FASP variants based on filters with different geometries and/or molecular weight cutoffs (namely, A10, A30, M10, and M30), two methods were SP3-based (with or without iST cleanup), and the last method was S-Trap. LC-MS/MS data were parsed to carry out a qualitative and quantitative comparison between the seven methods.

**Fig 2:**
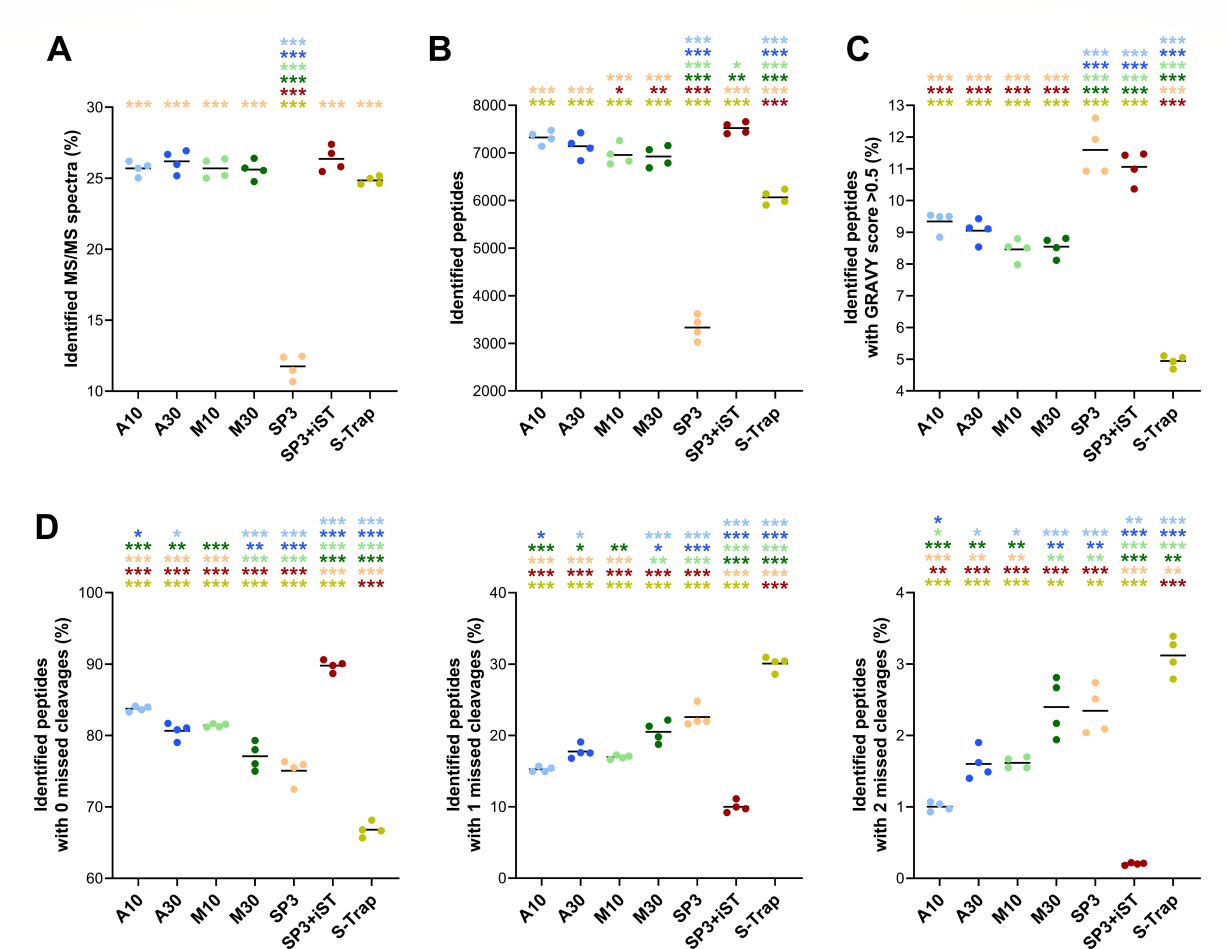
Peptide identification metrics measured in fecal samples processed using seven different low-throughput cleanup and digestion methods. Identification results regarding peptides of both microbial and human origin were considered. Each dot represents a technical replicate, whereas each method is marked with a different color. Black lines indicate the mean values for each method. Statistically significant differences between methods are indicated by asterisks (* =*P* < .05; ** =*P* < .01; *** =*P* < .001; one-way ANOVA), with the comparison being between the method represented under the asterisk and the method corresponding to the asterisk color. (**A**) Percentage of MS/MS spectra identified. (**B**) Number of peptides identified. (**C**) Percentage of identified peptides with a GRAVY score greater than 0.5 (i.e., hydrophobic peptides). (**D**) Percentage of identified peptides with 0 (left), 1 (middle), and 2 (right) missed cleavages.

As a preliminary metric, we examined the percentage of MS/MS spectra identified through a database search against gut microbiome and human sequences. As illustrated in Fig. 2A, all methods yielded comparable results, with an average of 25%–30% of MS/MS spectra identified, apart from the SP3 method, which reached significantly lower values (12% on average). To assess the degree of molecular diversity of the metaproteome profiles obtained with the different methods, the number of identified peptides per sample was measured. In other words, we computed the number of peptide sequences that were reliably matched to at least one MS/MS spectrum for each sample. SP3-iST provided the best performance (7522 peptides identified on average), closely followed by A10 and A30. M10 and M30 performed slightly worse, whereas S-Trap and, to a greater extent, SP3 led to the identification of significantly lower numbers of peptides (Fig. 2B). To evaluate the ability of the methods to preserve hydrophobic proteins, the percentage of peptides with a GRAVY score greater than 0.5 was calculated. As illustrated in Fig. 2C, the highest percentages were observed for SP3-based methods, with FASP-based methods occupying an intermediate position between these and the low-percentage S-Trap. The trypsin digestion efficiency was also investigated by examining the relative distribution of peptides with 0, 1, and 2 missed cleavages. As shown in Fig. 2D, SP3-iST exhibited the highest percentage of peptides with no missed cleavages (around 90%), with an almost negligible number of peptides with two missed cleavages. Conversely, the lowest percentage of peptides with no missed cleavages (less than 70%) was achieved by the S-Trap method, as well as the highest percentage of peptides with one or two missed cleavages (up to 30 and 3%, respectively). The remaining methods yielded between 75% and 85% of peptides with no missed cleavages, with A10 leading this intermediate group. The abundance and significance trends observed when considering only microbial peptide identifications (Fig. S1A) were nearly identical to those illustrated in Fig. 2.

Additionally, the PEP distribution at the PSM level was evaluated, with the objective of providing a measure of the false identification probability for each PSM (39). In other words, examining the PEP distribution in a data set provides a statistically grounded estimation of peptide identification reliability in that data set. As illustrated in Fig. S2, S-Trap, M10, and M30 exhibited the highest −log(PEP) values (based on the 1,000 best-scored PSMs identified in each of the four technical replicates analyzed per method), whereas SP3 demonstrated the least favorable performance.

**Fig 3:**
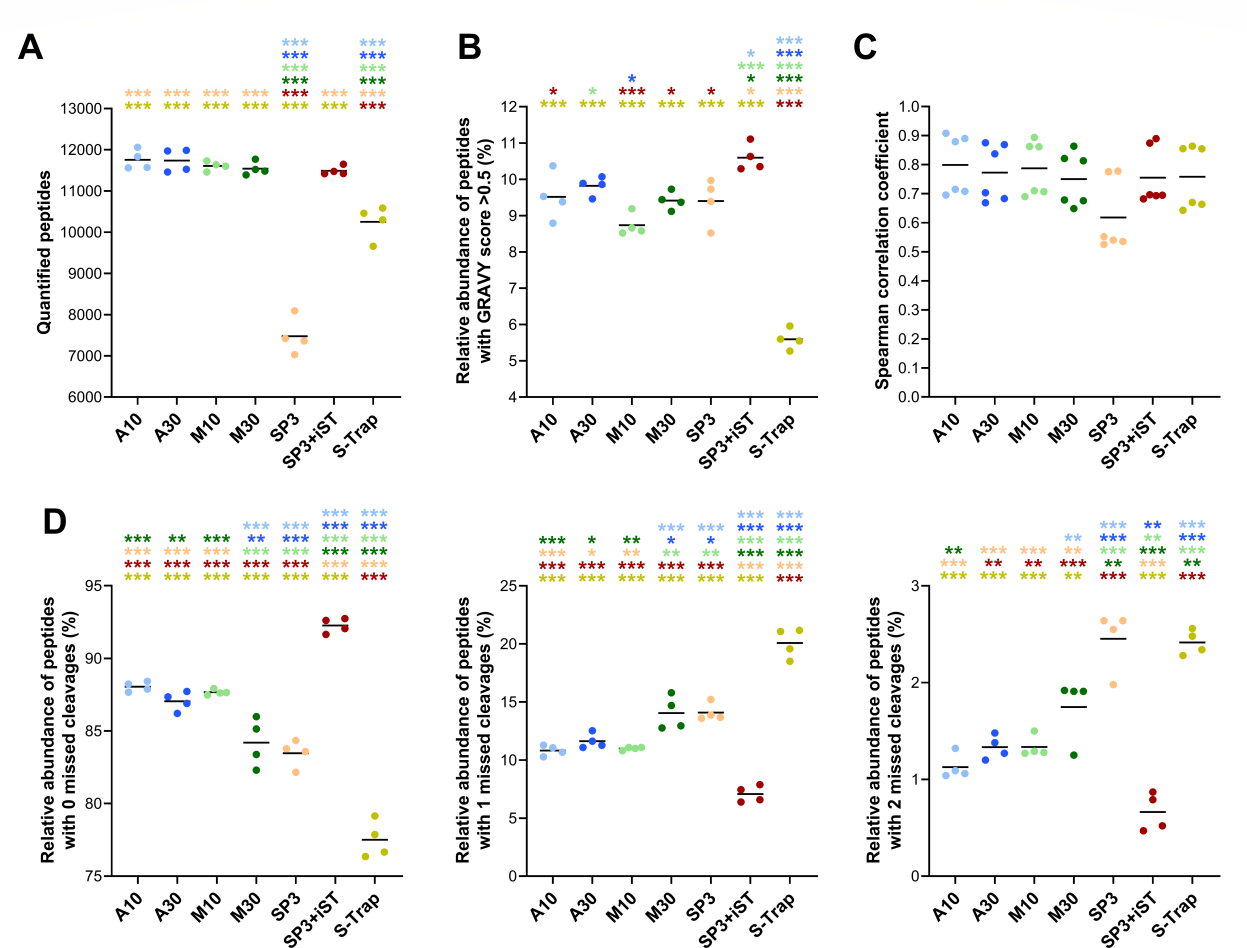
Peptide quantification metrics measured in fecal samples processed using seven different low-throughput cleanup and digestion methods. Label-free quantification data obtained from peptides of both microbial and human origin were considered. Each dot represents a technical replicate, whereas each method is marked with a different color. Black lines indicate the mean values for each method. Statistically significant differences between methods are indicated by asterisks (* =*P* < .05; ** =*P* < .01; *** =*P* < .001; one-way ANOVA), with the comparison being between the method represented under the asterisk and the method corresponding to the asterisk color. (**A**) Number of peptides quantified. (**B**) Relative abundance of peptides with a GRAVY score greater than 0.5 (i.e., hydrophobic peptides). (**C**) Correlation of peptide abundance values between technical replicates as measured by the Spearman correlation coefficient. (**D**) Relative abundance of peptides with 0 (left), 1 (middle), and 2 (right) missed cleavages.

To quantitatively compare the metaproteomic profiles, the LC-MS/MS data were further analyzed according to an MS1-based label-free quantification approach, based on the integrated area of the chromatographic peak associated with each peptide (as further detailed and discussed in the Materials and Methods and Discussion sections, respectively). No significant differences were observed between FASP-based methods and SP3-iST in terms of the number of peptides quantified per sample (all slightly below 12,000), whereas S-Trap and, to a greater extent, SP3 exhibited significantly lower values (Fig. 3A). Furthermore, SP3-iST and S-Trap were confirmed as the best- and worst-performing method, respectively, both in terms of the preservation of hydrophobic peptides (Fig. 3B) and the relative percentage of peptides with no missed cleavages (Fig. 3D), with the remaining methods occupying intermediate positions. In addition, we examined the level of reproducibility between technical replicates according to the Spearman correlation coefficient, finding no significant differences between the different methods (Fig. 3C).

**Fig 4:**
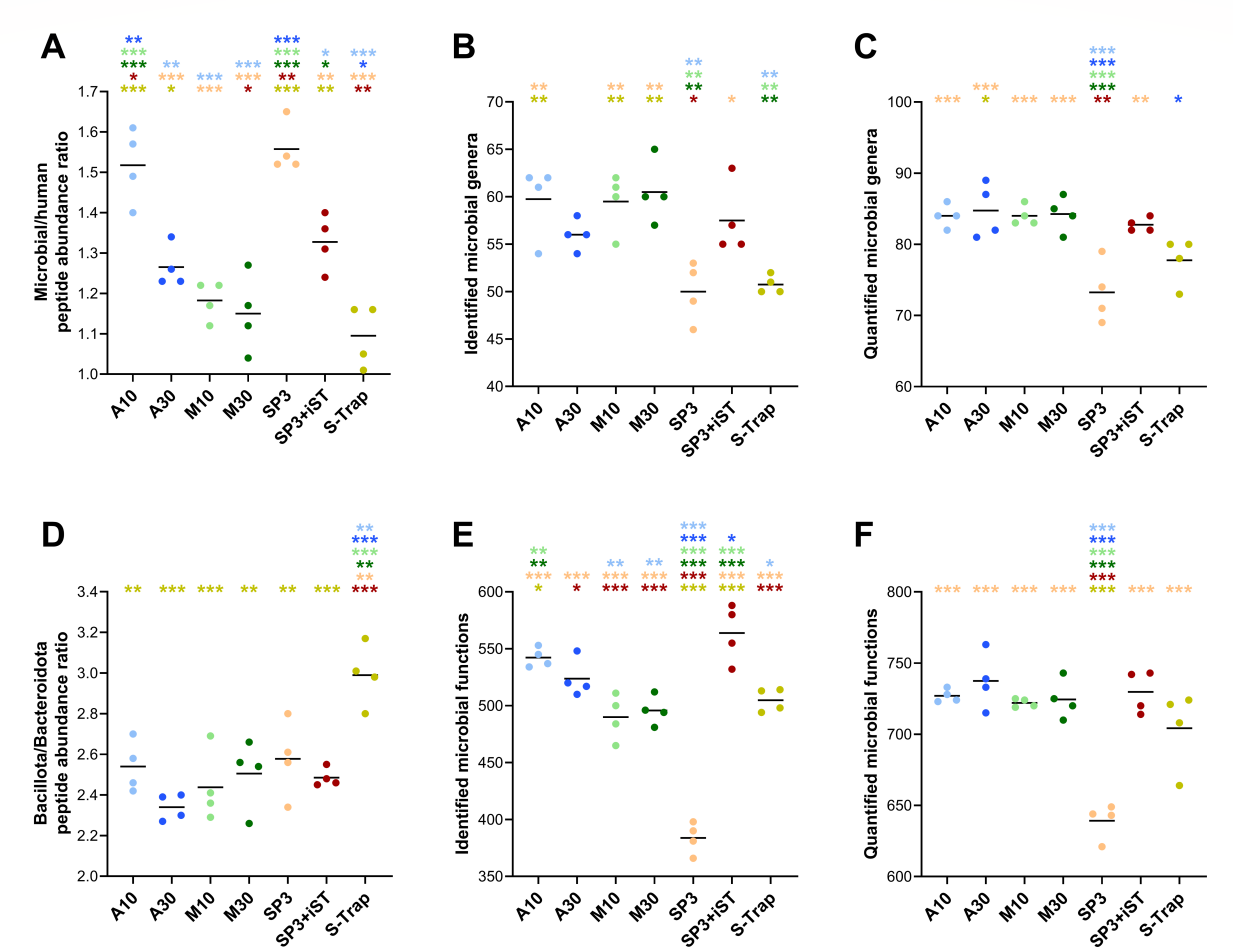
Microbial taxonomic and functional metrics measured in fecal samples processed using seven different low-throughput cleanup and digestion methods. Each dot represents a technical replicate, whereas each method is marked with a different color. Black lines indicate the mean values for each method. Statistically significant differences between methods are indicated by asterisks (* =*P* < .05; ** =*P* < .01; *** =*P* < .001; one-way ANOVA), with the comparison being between the method represented under the asterisk and the method corresponding to the asterisk color. (**A**) Ratio between relative abundances of microbial and human peptides. (**B**) Number of microbial genera identified per sample. (**C**) Number of microbial genera quantified per sample. (**D**) Ratio between the relative abundances of peptides assigned to Bacillota and Bacteroidota. (**E**) Number of microbial KEGG KO functions identified per sample. (**F**) Number of microbial KEGG KO functions quantified per sample.

When the scope was restricted to microbial peptides only, slight differences were observed in the relative abundance of peptides with a GRAVY score greater than 0.5 and of those with no missed cleavages (Fig. S1B), compared with the data obtained considering both human and microbial peptides (Fig. 3). In addition, the abundance ratio between peptides of microbial and human origin was calculated. As illustrated in Fig. 4A, this parameter exhibited significantly greater values for A10 and SP3 (over 1.5), indicating a higher proportion of microbial peptides compared with the other methods. Furthermore, label-free quantitative data related to microbial peptides were parsed to infer taxonomic and functional metrics. Specifically, taxonomic richness was measured according to the number of microbial genera identified (Fig. 4B) or quantified (Fig. 4C). In line with data at the peptide level, the highest richness values were achieved by FASP-based and SP3-iST methods. A common index related to the taxonomic structure of gut microbiota, the ratio between the abundances of Bacillota and Bacteroidota (formerly known as Firmicutes/Bacteroidetes ratio), was also calculated (Fig. 4D). Comparable values were observed for all methods (between 2.3 and 2.6 on average), except for S-Trap that exhibited relatively higher amounts of peptides encoded by Bacillota compared with the other protocols. Finally, functional richness was evaluated by measuring the total number of microbial KEGG KO functions identified (Fig. 4E) or quantified (Fig. 4F) in each sample. SP3-iST and A10 showed the highest functional richness values when considering identification data, whereas the results based on quantitative data were more comparable between methods, with the exception of the worst-performing SP3.

**Fig 5:**
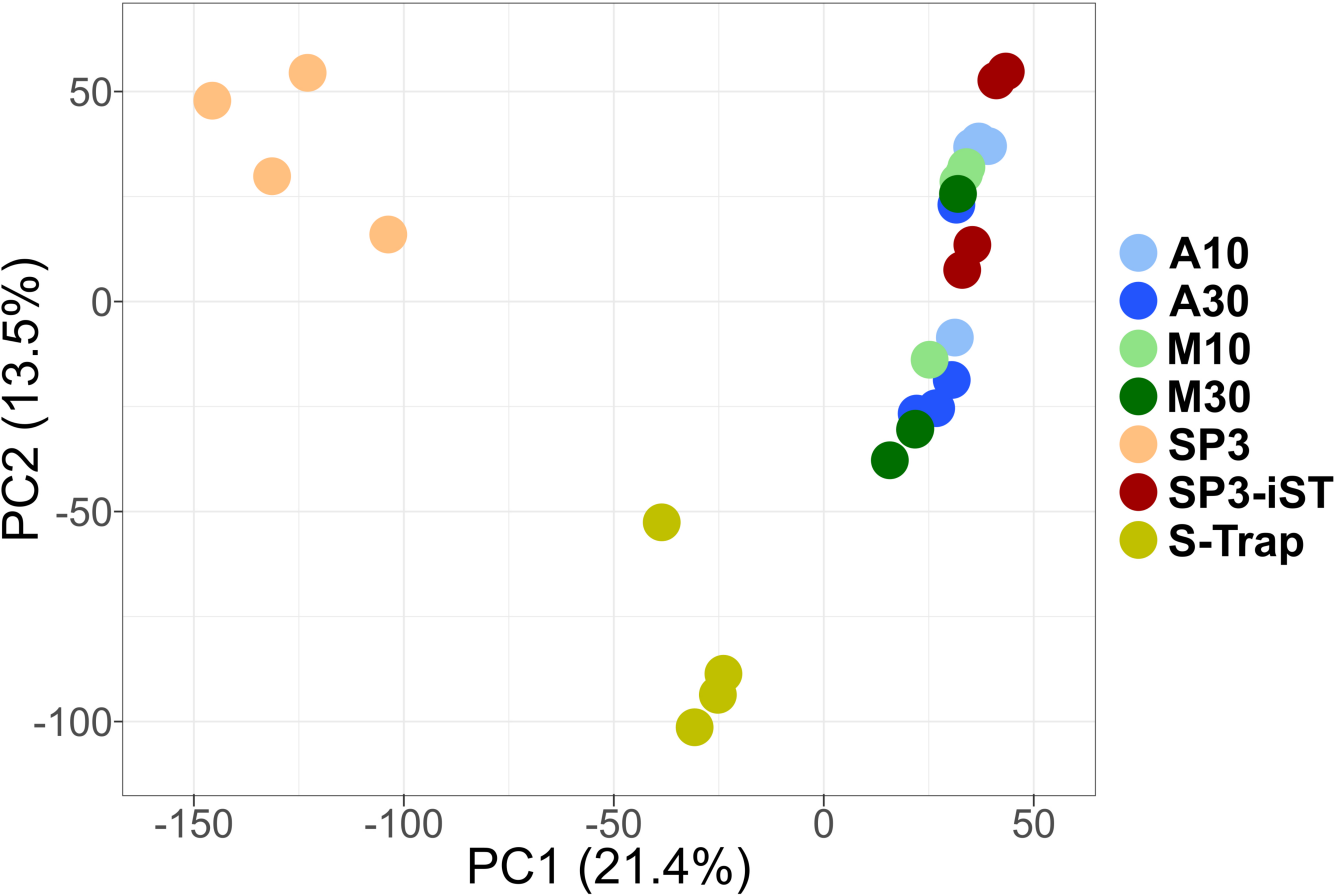
PCA plot based on peptide abundance values measured in fecal samples processed using seven different low-throughput cleanup and digestion methods. Label-free quantification data related to peptides of both microbial and human origin were considered. Each dot represents a sample (four technical replicates per method), whereas each method is marked with a different color. The percentages of variation explained by the first two components are shown in the x- and y-axes, respectively.

Furthermore, the beta diversity between samples was evaluated. As illustrated in the PCA plot of Fig. 5 and confirmed by PERMANOVA *P*-values (Table S1), the technical replicates of methods A10, A30, M10, M30, and SP3-iST exhibited a high degree of similarity, whereas the S-Trap and SP3 samples formed two distinct clusters, both significantly separated from the remaining sample groups.

Finally, we sought to determine if the metaproteomic data sets obtained by applying the seven low-throughput methods differed in terms of protein molecular weight distribution. To this end, we focused on extreme molecular weights (namely, <30 kDa and >200 kDa) and limited the analysis to human proteins (for which molecular weight information is reliable and validated, whereas incomplete sequences are likely to be contained in the collection of gut metagenomes used as microbial database in this study). Fig. S3 shows that no significant differences were observed when comparing FASP-based methods using filters with different molecular weight cutoffs (i.e., A10 vs A30 and M10 vs M30). Furthermore, the highest amounts of low- and high-molecular weight proteins were detected with FASP-based methods and SP3, respectively.

### Comparison of high-throughput sample preparation methods

In the second part of the study, we employed high-throughput protocols, suitable for large-scale metaproteomic studies and automated sample preparation systems. As a preliminary step, we conducted a search for devices and kits that were simultaneously: (i) based on the same principles of the previously compared low-throughput methods and (ii) available in a 96-well plate format. SP3 without iST was excluded due to the unsatisfactory performance achieved in the first part of the study. Among FASP-based methods, we included a patented plate based on the Microcon filter geometry (only available with a 10 kDa cutoff), along with a generic plate in two variants (with different molecular weight cutoffs). To our knowledge, no microplates based on the Amicon geometry were available on the market. A total of five high-throughput protein cleanup and digestion methods were compared, as schematized in Fig. 1B. To account for interindividual variability, fecal protein extracts from eight different subjects were tested in triplicate for each method. LC-MS/MS data were parsed to compare the five methods in qualitative and quantitative terms. The expected interindividual variability in gut microbiota structure and peptide identification yield was counterbalanced by the application of a paired-samples statistical test (namely, repeated measures ANOVA).

**Fig 6:**
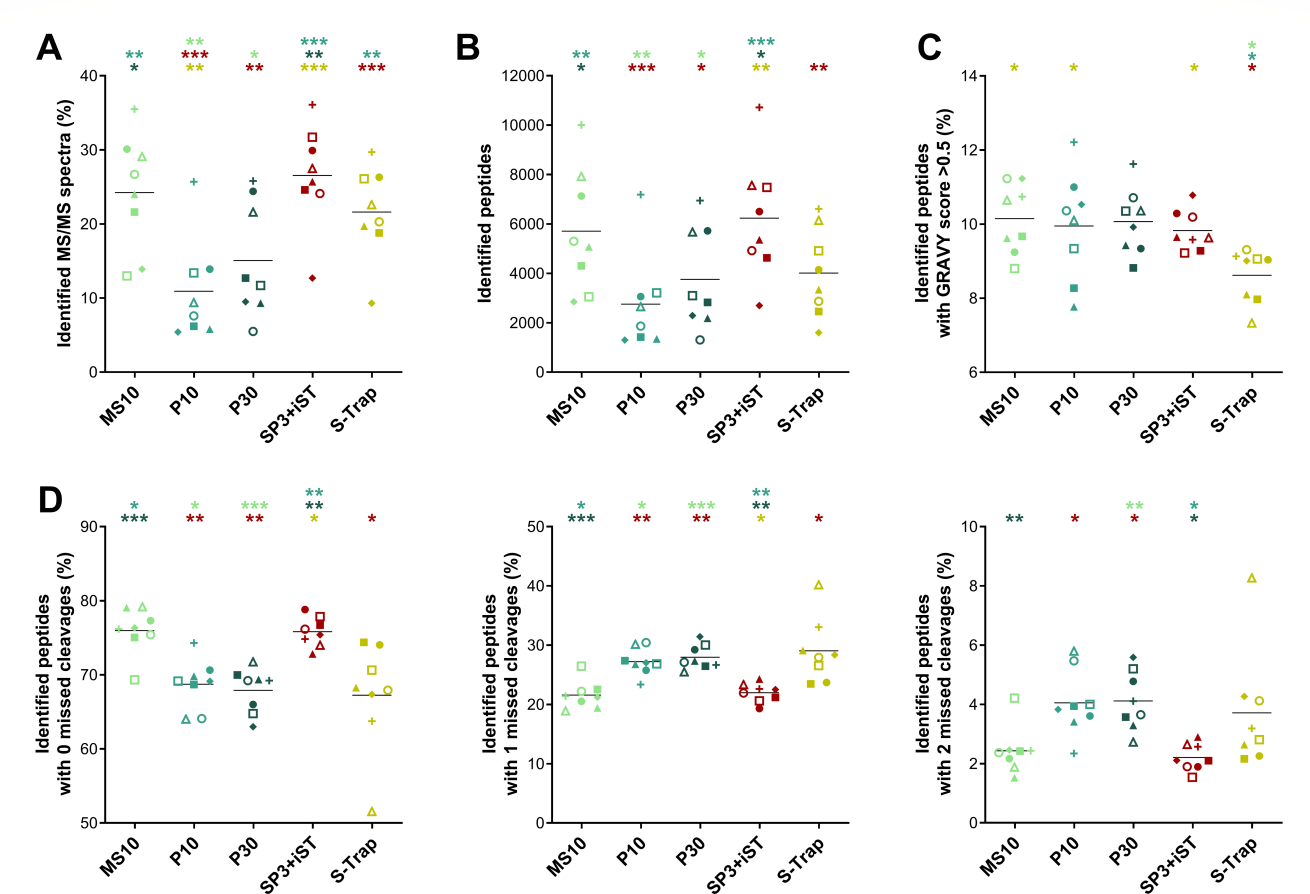
Peptide identification metrics measured in fecal samples processed using five different high-throughput cleanup and digestion methods. Identification results regarding peptides of both microbial and human origin were considered. Each shape represents a different subject (mean between three technical replicates), whereas each method is marked with a different color. Black lines indicate mean values for each method. Statistically significant differences between methods are indicated by asterisks (* =*P* < .05; ** =*P* < .01; *** =*P* < .001; repeated measures one-way ANOVA), with the comparison being between the method represented under the asterisk and the method corresponding to the asterisk color. (**A**) Percentage of MS/MS spectra identified. (**B**) Number of peptides identified. (**C**) Percentage of identified peptides with a GRAVY score greater than 0.5 (i.e., hydrophobic peptides). (**D**) Percentage of identified peptides with 0 (left), 1 (middle), and 2 (right) missed cleavages.

In terms of the percentage of MS/MS spectra identified (Fig. 6A) and the number of unique peptide sequences identified (Fig. 6B) upon database search against gut microbiota and human sequences, the results achieved using SP3-iST and MS10 were comparable, followed by S-Trap, P30, and P10. As expected, a notable degree of inter-individual variability was observed for both metrics, with up to a fourfold difference measured between subjects. With regard to the percentage of peptides with a GRAVY score greater than 0.5 (Fig. 6C), S-Trap yielded a significantly lower proportion of hydrophobic peptides, whereas the other methods exhibited comparable performance. As shown in Fig. 6D, SP3-iST and MS10 yielded comparable results also in terms of the percentage of peptides with no missed cleavages (76% on average), with the other methods leading to the identification of higher percentages of peptides with one or two missed cleavages.

In addition, as illustrated in Fig. S4, SP3-iST achieved the highest −log(PEP) values, indicating the statistical robustness of PSM assignments, followed by MS10, S-Trap, P30, and P10.

**Fig 7:**
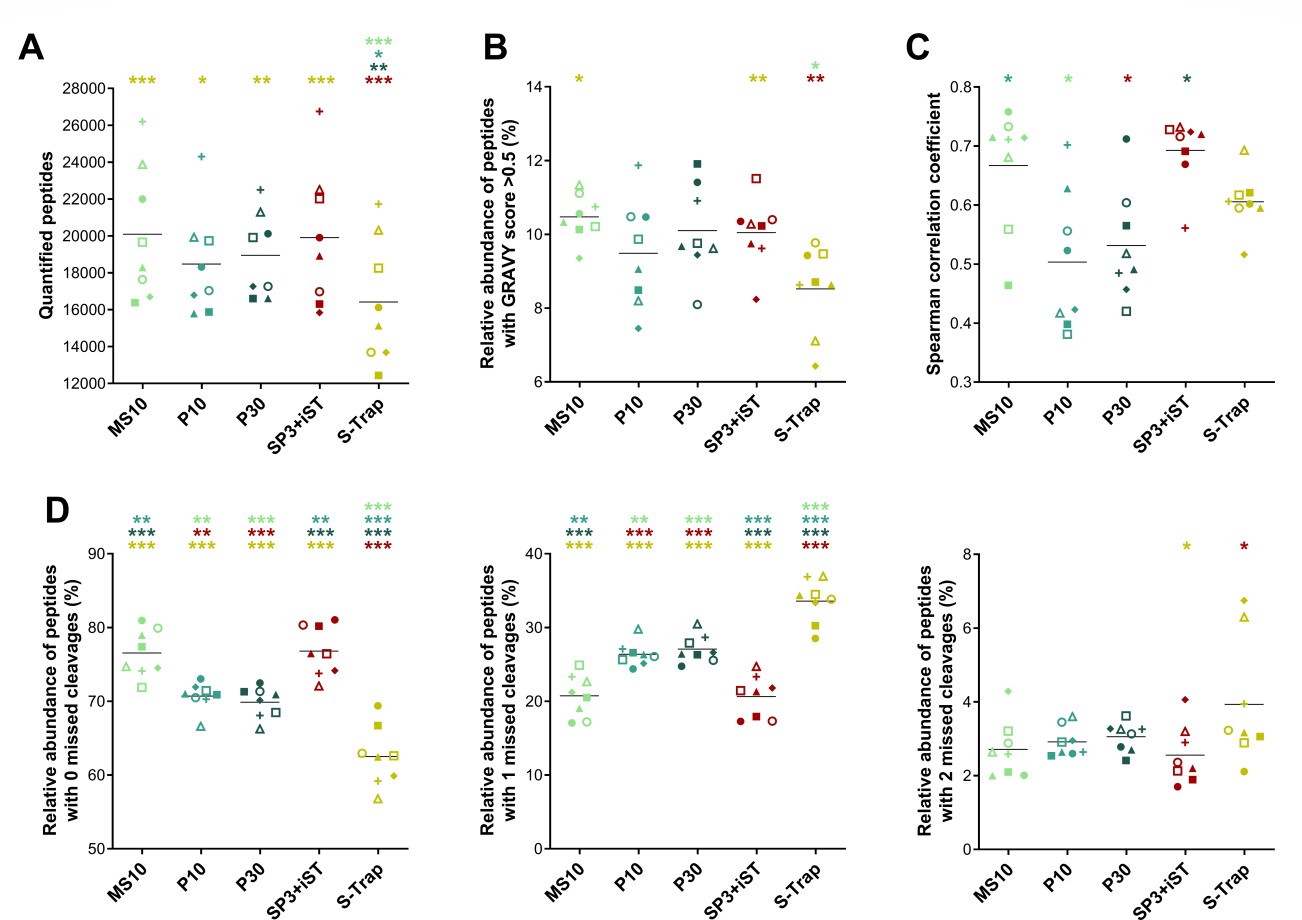
Peptide quantification metrics measured in fecal samples processed using five different high-throughput cleanup and digestion methods. Label-free quantification data obtained from peptides of both microbial and human origin were considered. Each shape represents a different subject (mean between three technical replicates), whereas each method is marked with a different color. Black lines indicate the mean values for each method. Statistically significant differences between methods are indicated by asterisks (* =*P* < .05; ** =*P* < .01; *** =*P* < .001; repeated measures one-way ANOVA), with the comparison being between the method represented under the asterisk and the method corresponding to the asterisk color. (**A**) Number of peptides quantified. (**B**) Relative abundance of peptides with a GRAVY score greater than 0.5 (i.e., hydrophobic peptides). (**C**) Correlation of peptide abundance values between technical replicates as measured by the (average) Spearman correlation coefficient. (**D**) Relative abundance of peptides with 0 (left), 1 (middle), and 2 (right) missed cleavages.

As previously described for low-throughput methods, metaproteomic profiles were quantitatively compared using the integrated area of the chromatographic peak associated with each peptide as a label-free quantitative measure. In terms of peptides quantified per sample (both in total and with a GRAVY score >0.5), comparable performances were reached by all methods, with the exception of S-Trap (Fig. 7A and B). Moreover, significantly higher values of the correlation coefficient between technical replicates (Fig. 7C) and the relative amount of peptide with no missed cleavages (Fig. 7D) were reached when using MS10 and SP3-iST compared with the other methods. These results indicate that MS10 and SP3-iST achieved the highest levels of both method reproducibility and digestion efficiency.

As already observed for the low-throughput methods, the results obtained when considering microbial and human peptides were similar to those related to microbial peptides only, both for identification (Fig. S5A) and label-free quantification (Fig. S5B) data.

**Fig 8:**
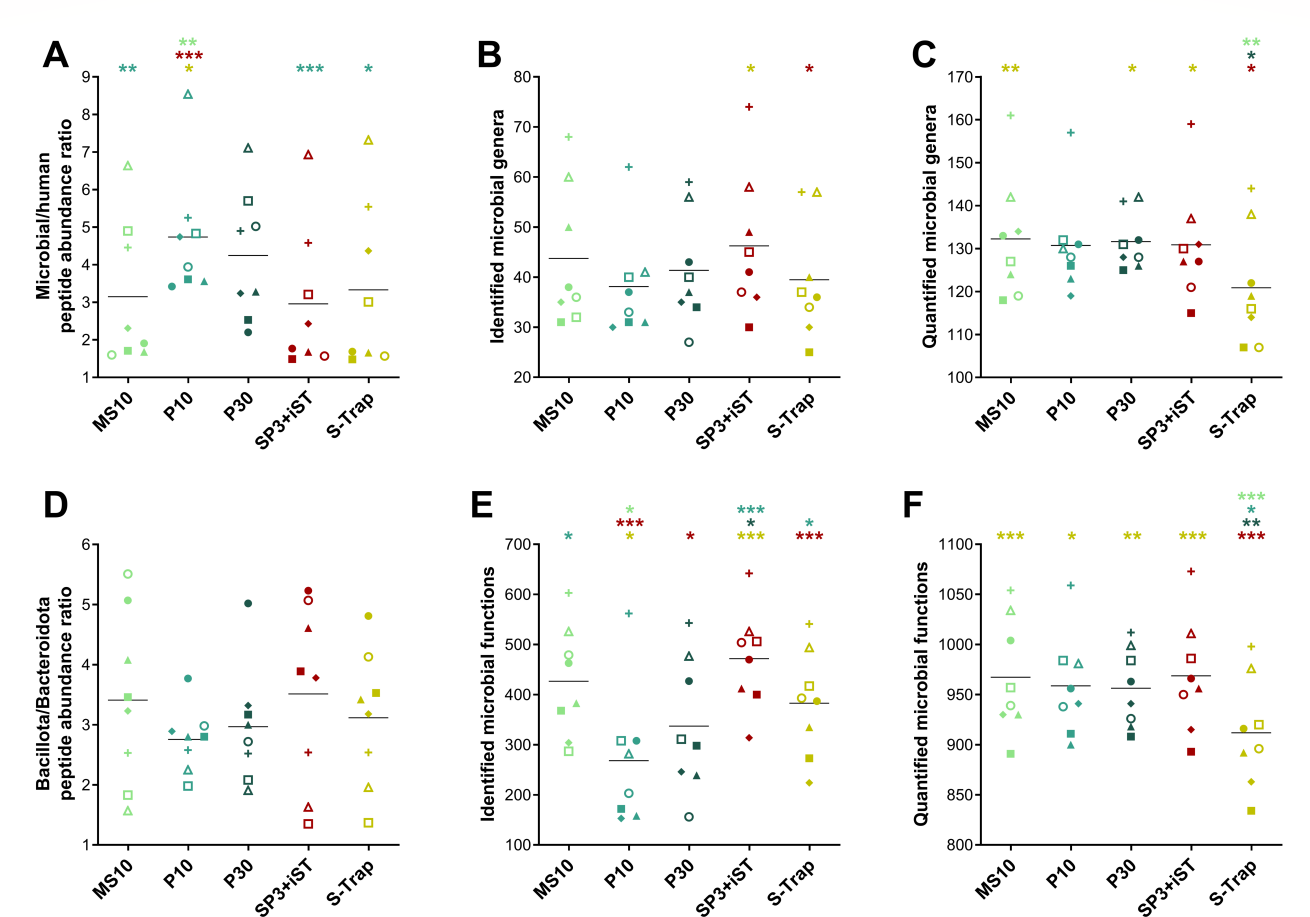
Microbial taxonomic and functional metrics measured in fecal samples processed using five different high-throughput cleanup and digestion methods. Each shape represents a different subject (mean between three technical replicates), whereas each method is marked with a different color. Black lines indicate the mean values for each method. Statistically significant differences between methods are indicated by asterisks (* =*P* < .05; ** =*P* < .01; *** =*P* < .001; repeated measures one-way ANOVA), with the comparison being between the method represented under the asterisk and the method corresponding to the asterisk color. (**A**) Ratio between the relative abundances of microbial and human peptides. (**B**) Number of microbial genera identified per sample. (**C**) Number of microbial genera quantified per sample. (**D**) Ratio between relative abundances of peptides assigned to Bacillota and Bacteroidota. (**E**) Number of microbial KEGG KO functions identified per sample. (**F**) Number of microbial KEGG KO functions quantified per sample.

As shown in Fig. 8A, the microbial/human peptide abundance ratio measured with the P10 method (4.7 on average) was significantly higher compared with MS10, SP3-iST, and S-Trap (3.2, 3.0, and 3.3 on average, respectively). Furthermore, microbial taxonomic and functional metrics were calculated. No clear differences were observed between high-throughput methods neither in taxonomic richness (Fig. 8B and C), except for slightly lower values measured for S-Trap based on label-free quantitative data, nor in the Bacillota/Bacteroidota ratio (Fig. 8D). Finally, SP3-iST exhibited the highest functional richness when considering identification data (Fig. 8E), whereas the results based on quantitative data were more comparable between methods, apart from S-Trap which reached lower values (Fig. 8F).

**Fig 9:**
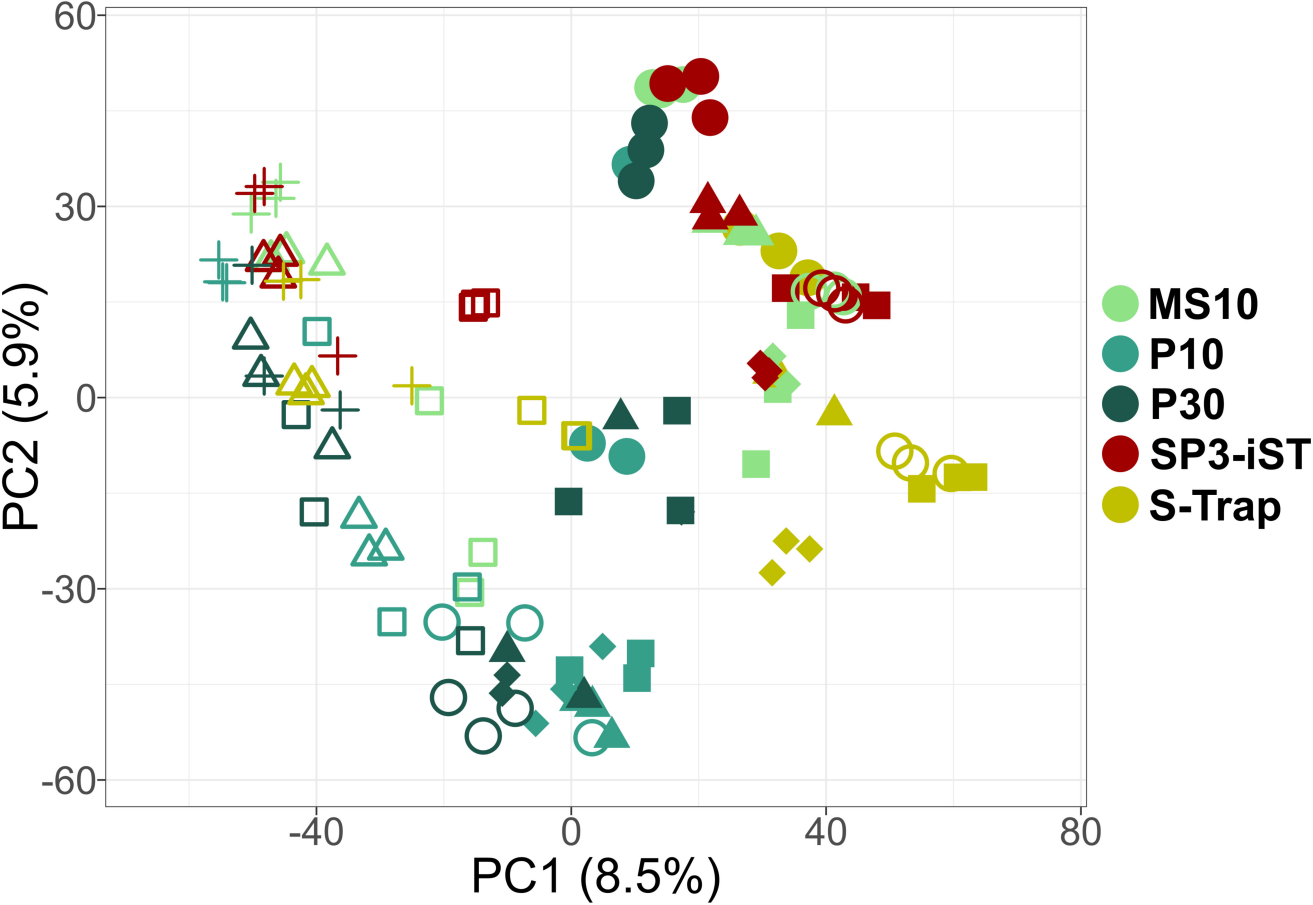
PCA plot based on peptide abundance values measured in fecal samples processed using five different high-throughput cleanup and digestion methods. Label-free quantification data related to peptides of both microbial and human origin were considered. Each shape represents a different subject (with three technical replicates per subject), whereas each method is marked with a different color. The percentages of variation explained by the first two components are shown in the x- and y-axes, respectively.

Beta diversity between samples was also assessed by PCA (Fig. 9). The separation between the five sample groups was found to be significant based on the results of the PERMANOVA analysis (Table S2). Nevertheless, the less significant *P*-value was obtained in the comparison between the SP3-iST and MS10 sample groups, indicating a higher degree of comparability.

Finally, a comparative analysis of protein molecular weight distribution was conducted by comparing the relative abundance values of human proteins with a molecular weight of less than 30 kDa and more than 200 kDa. As illustrated in Fig. S6, a slightly higher amount of low-molecular weight proteins was observed in MS10 compared with SP3-iST, whereas no significant differences were observed for high-molecular weight proteins.

## DISCUSSION

This study presents a comparison of automatable and high-throughput protein cleanup and digestion methods applied to human fecal samples. A wide array of protocols, both in low- and high-throughput formats, was evaluated in qualitative and quantitative terms. The relative distribution of proteins and peptides based on several physicochemical variables, including molecular weight, hydrophobicity, and number of missed cleavages, was also investigated. All the high-throughput protocols evaluated in this study are directly suitable for liquid and microplate handling devices available in laboratory automation systems. As previously stated, protocols involving protein precipitation or SDS-PAGE separation, although commonly used in fecal metaproteomics, were not included in the study due to their limited suitability for high-throughput and automated sample processing.

Given the distinctive features of stool, particularly in terms of molecular complexity, the results presented here cannot be generalized to other types of samples. Additionally, fecal samples themselves can vary considerably in texture, especially as a consequence of inflammatory or dysbiotic states. The stool samples used in this study were collected after a successful treatment for eradication of *H. pylori* infection. Although antibiotic therapies are known to cause profound alterations in the gut microbiota both in terms of total biomass and taxonomic composition, an almost complete reversal of these changes 30 days after the end of the therapy was demonstrated in a previous analysis of the fecal metaproteome in ten subjects belonging to the same cohort of this study (40). Based on these considerations, we anticipated that the samples analyzed in this study were not substantially different from those that can be obtained from healthy subjects. In addition, the impact of interindividual variability was considered by analyzing stool samples from eight different subjects through microplate-based methods. However, it cannot be ruled out that significantly different results might be observed if stool samples from individuals with acute or chronic intestinal dysbiosis were analyzed.

Moreover, the outcome of a comparison between different methods is expected to be significantly influenced by the reagents used. Indeed, markedly different data may have been obtained if other devices, reagents, or commercial kits, as well as different buffers for the preliminary protein extraction step, had been employed in this study. To minimize biases in protein digestion efficiency, we took care to use the same lot of trypsin for all methods. Nevertheless, we have chosen to adhere to the specifications outlined by each manufacturer regarding both the quantity of trypsin and the incubation conditions to avoid the risk of suboptimal digestion efficiency. It is also important to note that the selected methods differ substantially in both duration and cost. To facilitate comparisons, information regarding the approximate length and cost per sample of each protocol is provided in Table S3 (low-throughput methods) and S4 (high-throughput methods). Given the largely comparable efficiency of FASP-based and SP3-iST methods, the former is clearly preferable in terms of costs, whereas the latter in terms of overall protocol length. Every laboratory will consider the relative importance of each of the factor described here (namely, efficiency, time, and costs) based on its own specific requirements and constraints.

We have chosen to focus exclusively on two sample preparation steps (namely, protein cleanup and digestion) among all those constituting the complex workflow of a fecal metaproteomics experiment. With regard to the other two critical experimental phases (namely, sample storage and protein extraction), we chose to apply the standard protocols used in our laboratory for human feces. In particular, stool samples were immediately frozen and stored at −80°C until analysis, whereas protein extraction was based on an established combination of bead beating and heating/freezing steps (along with the use of an SDS/DTT-based buffer) (28). However, it is important to emphasize that both of these steps are essential to obtain a reliable and comprehensive metaproteome profile, as demonstrated by numerous studies (9, 10, 41–43). Therefore, specific efforts should be made in the future to standardize and automate both sample storage and protein extraction protocols.

The results of this study were presented both in terms of identification- and quantification-based data. According to the MS1-based label-free quantification approach employed here, a peptide that was not selected for fragmentation in a given sample can be quantified if its chromatographic peak aligns correctly with at least one peak, associated with the same mass and detected in another sample, for which a fragmentation spectrum is available. Despite a slight degree of false identifications, this approach was demonstrated to be capable of reaching remarkable levels of sensitivity, accuracy, and dynamic range (33). However, if a sample in which a given peptide was not identified is extrapolated from the data set and subjected to a separate quantification, that peptide will not even be quantified. In light of these considerations, we have presented both the identification and the quantification data as complementary information: the former provides qualitative insight into the results that can be obtained from a single sample (not considering the other samples/groups), whereas the latter delivers more quantitatively robust data about peptide abundance ratios between samples.

Our data indicate that the SP3-iST and FASP-based methods yielded the highest numbers of peptides identified and quantified. Additionally, significant differences were observed between the compared protocols in terms of the efficiency of protein digestion, the ability to preserve hydrophobic peptides and high molecular-weight proteins, and the reproducibility of the methods. Recently, the S-Trap, FASP (10 and 30 kDa cutoffs), and SP3 methods, all in microtube format, were applied to protein extracts obtained from mouse feces. According to the authors, the S-Trap method demonstrated superior performance in terms of the number of identifications, reproducibility, and digestion efficiency (25). One potential explanation for the discrepancy between our results and those reported by the authors is that laboratory mouse stool differs considerably in texture and composition, as well as in types and concentration of interfering molecules, from fecal samples collected from human subjects following an omnivorous diet. Similar method comparisons were performed with other human microbiota-associated samples, including urine (44), saliva (45), and sputum (46), with S-Trap and FASP being the best-performing protocols. Conversely, SP3 outperformed S-Trap in terms of peptide identifications when analyzing a laboratory-assembled microbial mixture (47). A further study analyzed lysates derived from a human cancer cell line and found that the performance of the sample preparation methods is strongly dependent on the starting protein amount. In particular, the authors observed that, when loading 20 μg of proteins, iST was the most effective method, followed by FASP and SP3. Conversely, at lower protein amounts (down to one microgram), SP3 still demonstrated high identification yields and reproducibility, whereas the performance of iST and FASP exhibited a moderate and dramatic decrease, respectively (48). Based on these data, it can be hypothesized that our results may have been significantly different if starting from lower amounts of fecal sample. In this study, although protein concentration was not explicitly measured in the fecal extracts, we expected approximately 20–40 μg of proteins to be present in the volume of sample loaded for protein cleanup and digestion, according to previous analyses carried out in our laboratory using the same extraction protocol (data not shown). Nevertheless, a more than decent quantity of proteins is typically obtained from human stool samples, making protein scarcity issues rather infrequent.

Furthermore, no clear differences were observed between the methods compared here with regard to the microbial/human peptide abundance ratio and other microbiota-specific metrics, including the Bacillota/Bacteroidota ratio and taxonomic/functional richness. This result was largely anticipated, given that all cleanup and digestion methods were applied to the same protein extracts and were all based (in contrast to extraction methods) on principles that are expected to exert a selective interaction with peptides based on their physicochemical characteristics, rather than on their specific microbial origin.

In perspective, it would be of interest to extend these comparative tests to data-independent analysis (DIA) approaches, which are emerging as high-performance MS methods also in the field of gut metaproteomics (49–52). With regard to this, (low-throughput) SP3 has been recently applied to mouse feces using a DIA workflow (26). Furthermore, the latest variants of some of the methods selected in this study, such as enhanced FASP (eFASP) (53, 54), ultrasonic-based FASP (55), and Solvent Precipitation SP3 (SP4) (56), might deserve evaluation, along with other recently developed protocols, including Sample Preparation by Easy Extraction and Digestion (SPEED) (57) and Miniprep-Assisted Proteomics (MAP) (58). In addition, the investigation might be extended to different types of SP3-suitable beads, even in various combinations between them, which may lead to significantly improved binding of specific categories of proteins and/or to better removal of interfering molecules.

In conclusion, this study provides comparative information that can be useful for the optimization and standardization of protein cleanup and digestion protocols in human fecal metaproteomics. Moreover, data concerning automatable high-throughput methods provide valuable indications for their application to large-scale studies targeting the human gut microbiota.

## Data Availability

Mass spectrometry proteomics data (along with identification data at the protein, peptide and PSM levels) have been deposited to the ProteomeXchange Consortium via the PRIDE (60) partner repository with the data set identifier PXD049294.

## SUPPLEMENTAL MATERIAL

Supplemental Figures and Tables msystems.00661-24-s0001.pdf Figures S1-S6; Tables S1-S4.

ASM does not own the copyrights to Supplemental Material that may be linked to, or accessed through, an article. The authors have granted ASM a non-exclusive, world-wide license to publish the Supplemental Material files. Please contact the corresponding author directly for reuse.
