# Supplementary material for: Benchmarking low- and high-throughput protein cleanup and digestion methods for human fecal metaproteomics
Source: mSystems. 2024 Jun 27;9(7):e00661-24. doi: 10.1128/msystems.00661-24 (PMC11265449; doi:10.1128/msystems.00661-24)
Supplement: Supplemental Figures and Tables — Figures S1-S6; Tables S1-S4. [file msystems.00661-24-s0001.pdf]

Benchmarking low- and high-throughput protein cleanup and digestion methods for human fecal metaproteomics

Alessandro Tanca[1,2], Maria Antonietta Deledda[1], Laura De Diego[1], Marcello Abbondio[1,2], Sergio Uzzau[1,2]

[1]*Department of Biomedical Sciences, University of Sassari, Sassari, Italy*

[2]*Unit of Microbiology and Virology, University Hospital of Sassari, Sassari, Italy*

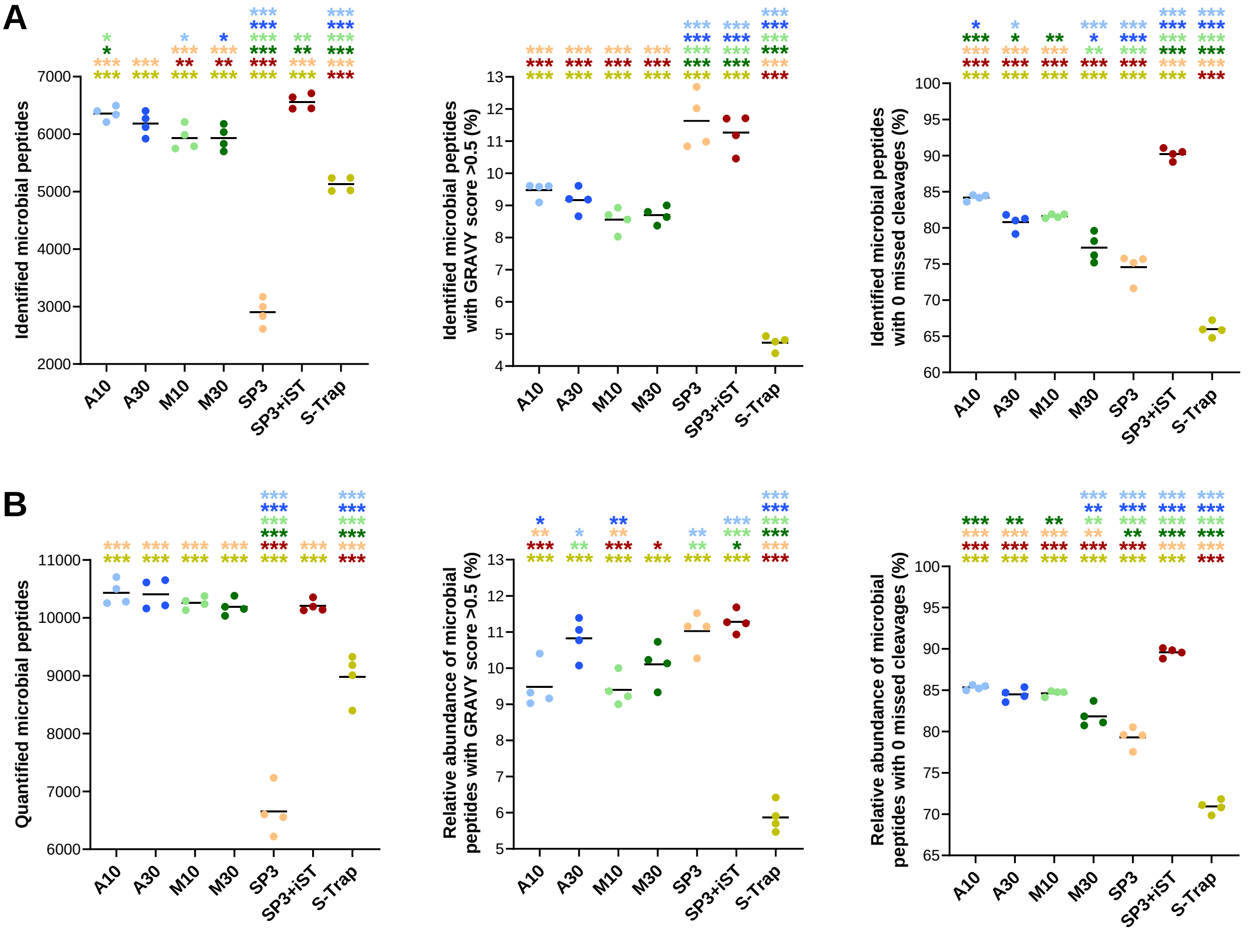

Figure S1. Microbial peptide identification and quantification metrics measured in fecal samples processed using seven different low-throughput cleanup and digestion methods. Each dot represents a technical replicate, while each method is marked with a different color. Black lines indicate the mean values for each method. Statistically significant differences between methods are indicated by asterisks (* = $p < 0.05$; ** = $p < 0.01$; *** = $p < 0.001$; one-way ANOVA), with the comparison being between the method represented under the asterisk and the method corresponding to the asterisk color. A) Number of identified microbial peptides (left); percentage of identified microbial peptides with a GRAVY score greater than 0.5 (middle); percentage of identified peptides with no missed cleavages (right). B) Number of quantified microbial peptides (left); relative abundance of microbial peptides with a GRAVY score greater than 0.5 (middle); relative abundance of peptides with no missed cleavages (right).

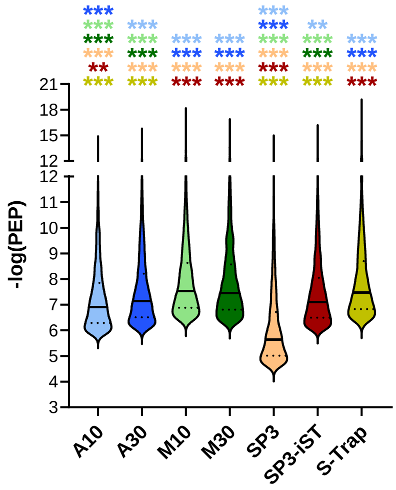

Figure S2. Violin plots showing posterior error probability (PEP) distribution, expressed as negative logarithm, measured in fecal samples processed using seven different low-throughput cleanup and digestion methods. For each method (four technical replicates), the graph reports PEP values calculated by Percolator for the 1000 best-ranked PSMs. Black solid and dotted lines indicate median and quartile values for each method, respectively. Statistically significant differences between methods are marked by asterisks (** = $p < 0.01$; *** = $p < 0.001$; one-way ANOVA), with the comparison being between the method represented under the asterisks and the method corresponding to the asterisk color.

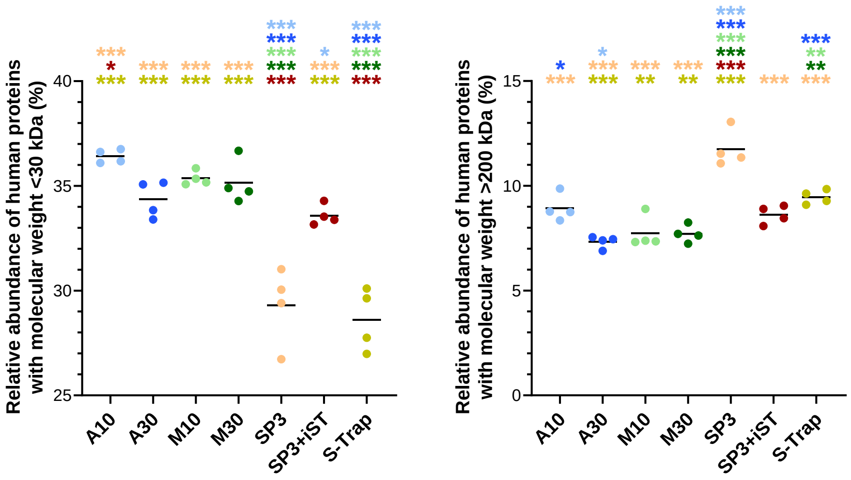

Figure S3. Protein quantification metrics measured in fecal samples processed using seven different low-throughput cleanup and digestion methods, with focus on extreme molecular weights. Percentage relative abundances of human proteins with molecular weight lower than 30 kDa (left) and higher than 200 kDa (right) are shown. Each dot represents a technical replicate, while each method is marked with a different color. Black lines indicate mean values for each method. Statistically significant differences between methods are marked by asterisk(s) (* = $p < 0.05$; ** = $p < 0.01$; *** = $p < 0.001$; one-way ANOVA), with the comparison being between the method represented under the asterisk and the method corresponding to the asterisk color.

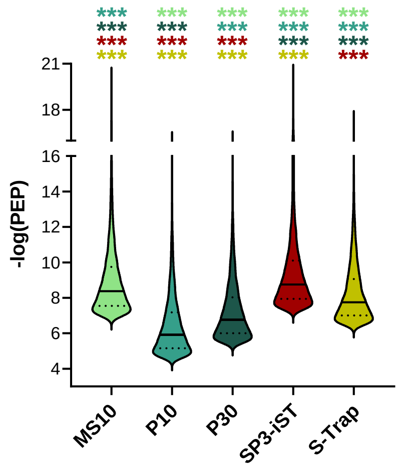

Figure S4. Violin plots showing posterior error probability (PEP) distribution, expressed as negative logarithm, measured in fecal samples processed using five different high-throughput cleanup and digestion methods. For each method (three technical replicates per subject), the graph reports PEP values calculated by Percolator for the 1000 best-ranked PSMs. Black solid and dotted lines indicate median and quartile values for each method, respectively. Statistically significant differences between methods are marked by asterisks (*** = $p < 0.001$; one-way ANOVA), with the comparison being between the method represented under the asterisks and the method corresponding to the asterisk color.

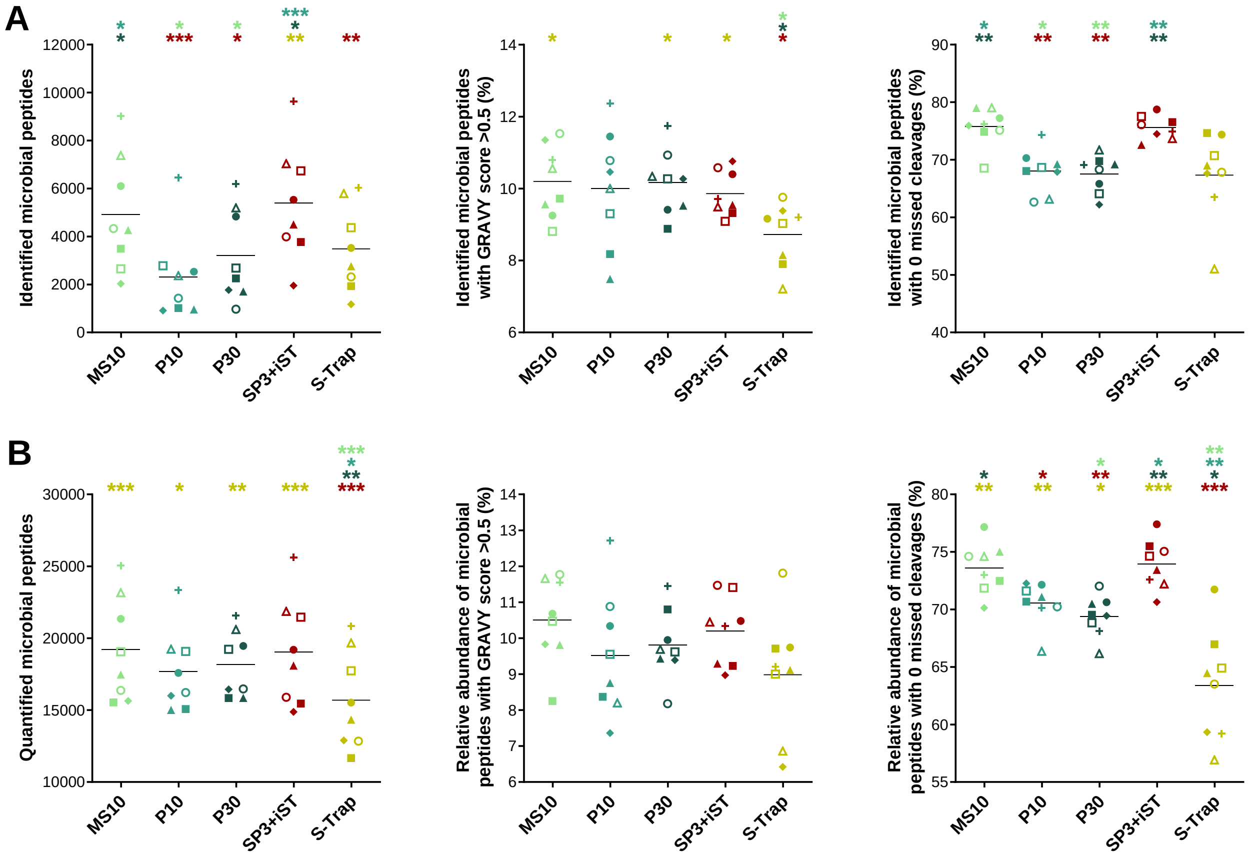

Figure S5. Microbial peptide identification and quantification metrics measured in fecal samples processed using five different high-throughput cleanup and digestion methods. Each shape represents a different subject (mean between three technical replicates), while each method is marked with a different color. Black lines indicate mean values for each method. Statistically significant differences between methods are indicated by asterisks (* = $p < 0.05$; ** = $p < 0.01$; *** = $p < 0.001$; repeated measures one-way ANOVA), with the comparison being between the method represented under the asterisk and the method corresponding to the asterisk color. A) Number of identified microbial peptides (left); percentage of identified microbial peptides with a GRAVY score greater than 0.5 (middle); percentage of identified peptides with no missed cleavages (right). B) Number of quantified microbial peptides (left); relative abundance of microbial peptides with a GRAVY score greater than 0.5 (middle); relative abundance of peptides with no missed cleavages (right).

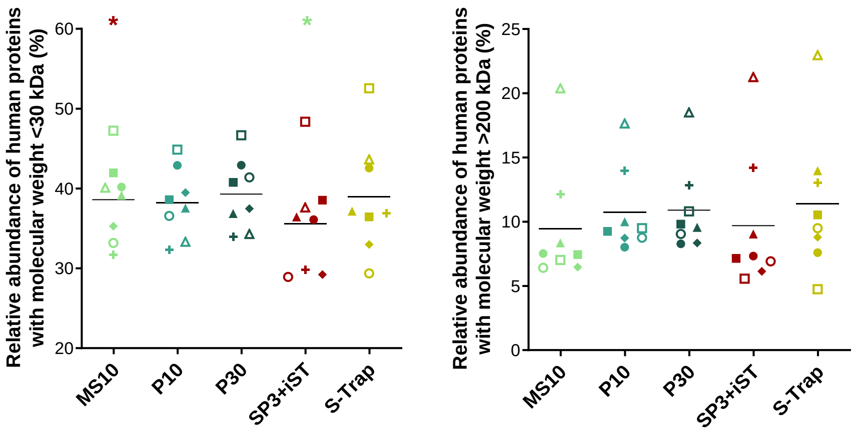

Figure S6. Protein quantification metrics measured in fecal samples processed using five different high-throughput cleanup and digestion methods, with focus on extreme molecular weights. Percentage relative abundances of human proteins with molecular weight lower than 30 kDa (left) and higher than 200 kDa (right) are shown. Each shape represents a different subject (mean between three technical replicates), while each method is marked with a different color. Black lines indicate mean values for each method. Statistically significant differences between methods are marked by an asterisk ($p < 0.05$; repeated measures one-way ANOVA), with the comparison being between the method represented under the asterisk and the method corresponding to the asterisk color.

Table S1. PERMANOVA p-values associated with the PCA plot shown in Figure 5, obtained after pairwise comparison of low-throughput methods

Method 1	Method 2	p-value
A10	A30	0.053
A10	M10	0.460
A10	M30	0.060
A10	SP3	**0.026**
A10	SP3-iST	0.775
A10	S-Trap	**0.033**
A30	M10	0.062
A30	M30	0.473
A30	SP3	**0.034**
A30	SP3-iST	0.083
A30	S-Trap	**0.031**
M10	M30	0.063
M10	SP3	**0.034**
M10	SP3-iST	0.326
M10	S-Trap	**0.024**
M30	SP3	**0.028**
M30	SP3-iST	0.105
M30	S-Trap	**0.029**
SP3	SP3-iST	**0.025**
SP3	S-Trap	**0.018**
SP3-iST	S-Trap	**0.021**

Table S2. PERMANOVA p-values associated with the PCA plot shown in Figure 9, obtained after pairwise comparison of high-throughput methods

**Group 1**	**Group 2**	**p-value**
MS10	P10	**0.001**
MS10	P30	**0.001**
MS10	SP3-iST	**0.012**
MS10	S-Trap	**0.001**
P10	P30	**0.003**
P10	SP3-iST	**0.001**
P10	S-Trap	**0.001**
P30	SP3-iST	**0.001**
P30	S-Trap	**0.001**
SP3-iST	S-Trap	**0.001**

Table S3. Approximate duration and cost of the low-throughput methods compared in this study

Method code	Protocol duration (min)[#]	Digestion duration (h)[$]	Cost per sample (€)[§]
**A10**	140	1-ON	6
**A30**	80	1-ON	6
**M10**	140	1-ON	5
**M30**	80	1-ON	5
**SP3**	50	1-3	15
**SP3-iST**	75	1-3	45
**S-Trap**	30	1-ON	27

[#] The duration values were calculated based on the specifications provided by the manufacturers of the commercial kits or the original developers of the protocols. The incubation time for protein digestion was not included in the calculation, as it is provided in a separate column.

[$] The upper and lower extremes of the time range provided by the kit manufacturers or protocol developers are shown. ON, overnight.

[§] Costs are based on the selling prices registered in Italy during 2022.

Table S4. Approximate duration and cost of the high-throughput methods compared in this study

Method code	Protocol duration (min)#	Digestion duration (h)$	Cost per sample (€)§
**MS10**	135	1-ON	3
**P10**	65	1-ON	0.5
**P30**	65	1-ON	0.5
**SP3-iST**	50	1-3	30
**S-Trap**	30	1	5

The duration values were calculated based on the specifications provided by the manufacturers of the commercial kits or the original developers of the protocols. The incubation time for protein digestion was not included in the calculation, as it is provided in a separate column.

$ When appropriate, the upper and lower extremes of the time range provided by the kit manufacturers or protocol developers are shown. ON, overnight.

§ Costs are based on the selling prices registered in Italy during 2022.
